# Thiopurine withdrawal during sustained clinical remission in inflammatory bowel disease: relapse and recapture rates, with predictive factors in 237 patients

**DOI:** 10.1111/apt.12980

**Published:** 2014-10-06

**Authors:** N A Kennedy, R Kalla, B Warner, C J Gambles, R Musy, S Reynolds, R Dattani, H Nayee, R Felwick, R Harris, S Marriott, S M Senanayake, C A Lamb, H Al-Hilou, D R Gaya, P M Irving, J Mansfield, M Parkes, T Ahmad, J R F Cummings, I D Arnott, J Satsangi, A J Lobo, M Smith, J O Lindsay, C W Lees

**Affiliations:** * Gastrointestinal Unit, Western General HospitalEdinburgh, UK; † Gastroenterology, Royal Sussex County HospitalBrighton, UK; ‡ Gastroenterology and Liver Unit, Royal Hallamshire HospitalSheffield, UK; § Gastroenterology, Barts Health NHS TrustLondon, UK; ¶ Gastroenterology, Southampton General HospitalSouthampton, UK; ** University of Exeter Medical School and Royal Devon and Exeter NHS Foundation Trust; †† Gastroenterology Research Unit, Addenbrooke's HospitalCambridge, UK; ‡‡ Institute of Cellular Medicine, Newcastle UniversityNewcastle upon Tyne, UK; §§ Gastroenterology, Guy's and St Thomas' NHS Foundation TrustLondon, UK; ¶¶ Gastroenterology, Glasgow Royal InfirmaryGlasgow, UK; *** Gastroenterology, Royal Victoria InfirmaryNewcastle upon Tyne, UK

## Abstract

**Background:**

Thiopurines (azathioprine and mercaptopurine) remain integral to most medical strategies for maintaining remission in Crohn's disease (CD) and ulcerative colitis (UC). Indefinite use of these drugs is tempered by long-term risks. While clinical relapse is noted frequently following drug withdrawal, there are few published data on predictive factors.

**Aim:**

To investigate the success of planned thiopurine withdrawal in patients in sustained clinical remission to identify rates and predictors of relapse.

**Methods:**

This was a multicentre retrospective cohort study from 11 centres across the UK. Patients included had a definitive diagnosis of IBD, continuous thiopurine use ≥3 years and withdrawal when in sustained clinical remission. All patients had a minimum of 12 months follow-up post drug withdrawal. Primary and secondary end points were relapse at 12 and 24 months respectively.

**Results:**

237 patients were included in the study (129 CD; 108 UC). Median duration of thiopurine use prior to withdrawal was 6.0 years (interquartile range 4.4–8.4). At follow-up, moderate/severe relapse was observed in 23% CD and 12% UC patients at 12 months, 39% CD and 26% UC at 24 months. Relapse rate at 12 months was significantly higher in CD than UC (*P* = 0.035).

Elevated CRP at withdrawal was associated with higher relapse rates at 12 months for CD (*P* = 0.005), while an elevated white cell count was predictive at 12 months for UC (*P* = 0.007).

**Conclusion:**

Thiopurine withdrawal in the context of sustained remission is associated with a 1-year moderate-to-severe relapse rate of 23% in Crohn's disease and 12% in ulcerative colitis.

## Introduction

Thiopurines have been in clinical use for 50 years and remain the backbone of maintenance strategies for IBD, either as monotherapy or in combination with an anti-tumour necrosis factor agent.^1^ Azathioprine (AZA) and its metabolite mercaptopurine (MP) are effective in maintaining clinical remission in patients with Crohn's disease (CD) and ulcerative colitis (UC).^2^–^8^ Around 10–28% of patients report side effects (most commonly nausea) of which 50–80% will discontinue the drug as a result.^9^,^10^ Thiopurines have a narrow therapeutic window and carry a risk of dose-dependent myelosupression^4^,^9^,^11^,^12^ and hepatotoxicity.^10^,^13^ A subset of the population who carry two loss of function thiopurine methyltransferase (TPMT) alleles have the greatest risk of myelosupression and serious adverse events.^14^ Continuous use of thiopurines has also been linked with malignancies such as lymphoma and non-melanoma skin cancer.^15^,^16^ A large prospective study of 19 486 IBD patients showed incidence rates of non-melanoma skin cancer for current and previous AZA use at 0.66/1000 and 0.38/1000 patient years respectively (age < 50) and a cumulative increase with age.^15^ Beaugerie showed an incidence rate of 0.9/1000 patient years for lymphoma in those receiving AZA (*n* = 19 486), casting doubt on its long-term safety.^16^ These long-term risks make clinicians and patients wary about indefinite use of thiopurines despite the risk of relapse on withdrawal.

The relapse rates after stopping thiopurines have been reported in CD at 21–41% at 1 year with a cumulative increase to 61–85% at 5 years.^3^,^17^–^21^ In UC, one randomised controlled trial and three retrospective studies showed relapse rates of 35–77% at 1 year and 65–75% at 5 years.^9^,^22^–^24^ However, most of these studies had patients on treatment for a short period of time (Table1) and perhaps therefore overestimate the risk of disease relapse in patients who are in sustained clinical remission.

**Table 1:** Summary of AZA/MP withdrawal studies

Study	Design Study cohort	Number of patients studied following thiopurine withdrawal	Drug	Duration of thiopurine (months)	Follow-up (months)	Relapse rate		Factors predictive of relapse
O'Donoghue (1978)^3^	RCT CD	51	AZA	>24	6	1 year: Control 41%; AZA 5%		
Lemmann (2005)^19^	RCT CD	43	AZA	>42	18	18 months: Control 21.3%; AZA 7.9%		CRP Hb Time without steroids
Bouhnik (1996)^17^	Retrospective CD	42	AZA or MP	31 (median)	60	1 year: 38% 3 years: 61% 5 years: 75%		Male gender Age <31 years Remission<4 years
Kim (1999)^20^	Audit CD	36	MP	>6	60	1 year: 36% 2 years: 71% ≥3 years: 85%		Younger age Higher dose of 6MP during remission
Treton (2009)^18^	Open label CD	66	AZA	68 (median)	60	1 year: 14% 3 years: 52.8% 5 years: 62.7%		CRP ≥20 g/L Hb <120 g/L Neutrophils ≥4.0 × 10^9^/L
Fraser (2002)^9^	Retrospective case series CD and UC	CD – 79 UC – 143	AZA	24 (mean)	60	1 year: 3 years: 5 years:	CD/UC 37%/37% 66% 75%/75%	
Sokol (2010)^31^	Audit CD	47	AZA	>42	60	2 years: 57% 5 years: 73.3%		Male Non-smoking
Hawthorne (1992)^24^	RCT UC	34	AZA	21 (mean)	12	1 year: Control 59%; AZA 35%		
Lobel (2004)^22^	Retrospective review UC	22	MP	45 (median)	40	1 year: 77% 2 years: 100%		
Cassinotti (2009)^23^	Retrospective review UC	127	AZA	47 (median)	60	1 year: 35% 3 years: 59% 5 years: 65%		Shorter duration of AZA (in remission)

We aimed to examine relapse rates following thiopurine withdrawal along with predictive factors and the success of recapture in a large group of patients with at least 3 years of continuous thiopurine therapy for CD or UC.

## Methods

### Study design

A retrospective multi-centre clinical audit was performed with patients identified from 11 IBD centres across the UK. Detailed case note review was performed in all patients using a standardised, pre-designed proforma. Data were collected for patient demographics including age, sex, weight, smoking status, age at diagnosis and date of diagnosis. Details of drug therapy included the type of thiopurine used, start date, initial dose and maximum dosage, age at withdrawal and any dose tapering at withdrawal, plus concomitant medications. Details of parameters at withdrawal included Montreal classification and behaviour, laboratory markers [C-reactive protein (CRP), haemoglobin, white cell count, platelets, albumin], endoscopic findings and reasons for withdrawal. Relapse was recorded including any change in drug therapy or reintroduction of thiopurines. Patients were identified by searching IBD databases and/or clinic lists of those attending out-patient IBD clinics to reduce the risk of bias from physicians recalling only those patients who had relapsed.

### Inclusion criteria

Patients had a definitive diagnosis of UC or CD and continuous thiopurine use for at least 35 months. They were in clinical remission at the time of drug withdrawal as defined by physician global assessment and no use of corticosteroids within the preceding 6 months. The minimum follow-up time following withdrawal was 12 months (or moderate–to-severe relapse within 12 months). Patients were excluded if they were on concomitant anti-TNF therapy at the point of thiopurine withdrawal.

Disease relapse was defined by severity and categorised as mild, moderate or severe. Mild relapse was defined by the use of topical treatments or commencement or dose increase of oral 5-aminosalicylate (5-ASA) while moderate relapse was defined by the use of oral steroids or recommencement of thiopurine. Admission to hospital, surgery, use of intravenous corticosteroids or commencement of anti-TNF was considered a severe relapse.

At study design, primary end-point was defined as moderate-to-severe relapse at 12 months while secondary end-point was moderate-to-severe relapse at 24 months.

### Statistical analysis

Data were analysed using Microsoft Excel 2010 (Microsoft, Redmond, WA, USA) and R 3.1.1 (R Foundation for Statistical Computing, Vienna, Austria). Continuous data are presented as medians and interquartile ranges and were analysed using a Mann–Whitney *U*-test. Categorical data are presented as numbers and percentages and were analysed using χ^2^ or Fisher's exact tests as appropriate. Survival analysis was done using Kaplan–Meier analysis in the survival package in R. Patients was censored at the point of most recent follow-up. Estimates of relapse rates for each severity category over time were generated from the overall survival function and the proportion of relapses of that category to that time point. For analysis of predictive factors, each factor was analysed in those patients for whom those data were available. Patients with additional reasons for withdrawal that could potentially have influenced laboratory parameters were excluded from analysis of these parameters.

Multivariable analysis was performed using Cox Proportional Hazards. Backward stepwise regression was used to select variables for the final model. Variables that did not lead to a lower Akaike information criterion (AIC) were excluded in a stepwise manner, and finally variables whose hazard ratio had a 95% confidence interval that crossed one were excluded. Continuous data were then converted to categorical data by finding the thresholds that gave the lowest AIC for the fitted model.

## Results

Across all centres, 264 patients were submitted. 27 were excluded, for reasons detailed in Table S1, leaving 237 patients, 129 with CD and 108 with UC, in the primary analysis (Table2; breakdown by study centre in Table S2). The median duration of thiopurine use prior to drug withdrawal was 6.0 years (IQR 4.4–8.2) for CD and 5.8 years (IQR 4.5–8.5) for UC. The median follow-up post drug withdrawal in those without relapse was 32 months (IQR 24–51) for CD and 36 months (IQR 21–52) for UC. Median CRP was 4.0 mg/L (IQR 2.5–6.0) in CD and 2.5 mg/L (IQR 2.5–4.0) in UC (Table3).

**Table 2:** Study demographics, Montreal classification and disease behaviour for patients in clinical remission on thiopurines

Variable	Crohn's disease, *n* = 129	Ulcerative colitis, *n* = 108
Females (%)	76 (59.8%)	42 (39.6%)
Median (IQ range) age at withdrawal/years	38 (28–48)	42 (33–58)
Current smokers (%)*	23 (19.2%)	4 (4.3%)
Median (IQR) duration thiopurine use/years	6.0 (4.4–8.2)	5.8 (4.4–8.5)
Range duration thiopurine use/years	2.9–18.7	2.9–18.0
Median (IQR) peak AZA dose/mg	125 (100–150)	150 (112–150)
Median (IQR) duration follow-up in those without relapse/months	31.7 (23.9–50.8)	36.0 (20.6–52.2)
Median year stopped AZA (range)	2008 (1980–2012)	2008 (1999–2011)
Montreal location†		
L1 ± L4	29/123 (23.6%)	
L2 ± L4	48/123 (39.0%)	
L3 ± L4	44/123 (35.8%)	
L4	2/123 (1.6%)	
Montreal behaviour‡		
B1	88/123 (71.5%)	
B2	16/123 (13.0%)	
B3	19/123 (15.4%)	
Montreal extent‡		
E1		23/97 (23.7%)
E2		26/97 (26.8%)
E3		48/97 (49.5%)
5ASA at time of withdrawal	40 (31.0%)	83 (76.1%)

* Smoking status unknown in 23 patients.

† Montreal location and behaviour unknown in six patients

‡ Montreal extent unknown in 11 patients.

**Table 3:** Blood parameters for Crohn's disease and ulcerative colitis cohort at the time of thiopurine withdrawal

Test	Crohn's disease		Ulcerative colitis	
	Number of patients	Median (IQR)	Number of patients	Median (IQR)
Haemoglobin (g/L)*	107	150 (141–158)	94	148 (140–155)
White cell count (10^9^/L)	107	6.4 (5.3–8.4)	94	6.0 (4.8–7.0)
Platelets (10^9^/L)	105	266 (220–343)	92	260 (213–312)
CRP (mg/L)	81	4.0 (2.5–6.0)	65	2.5 (2.0–4.0)
Faecal calprotectin (μg/g)	6	36 (27–71)	2	71 (56–86)
Albumin (g/L)	87	43 (41–46)	73	45 (42–47)

Hb, Haemoglobin; WCC, White cell count; Plt, Platelets; CRP, C-reactive protein.

* Hb for females was scaled to male range to allow for comparison across sexes.

All patients were in sustained clinical remission at the time of thiopurine withdrawal; 35/237 (22 CD; 13 UC) had an additional trigger for drug cessation (Table S3). Thiopurines were tapered prior to withdrawal in 87 patients (37%). Data on length of taper were available in 48 of these patients, with a median duration of 12 weeks (IQR 8–26).

### Disease relapse and predictive factors: univariable analysis

**Figure 1:**
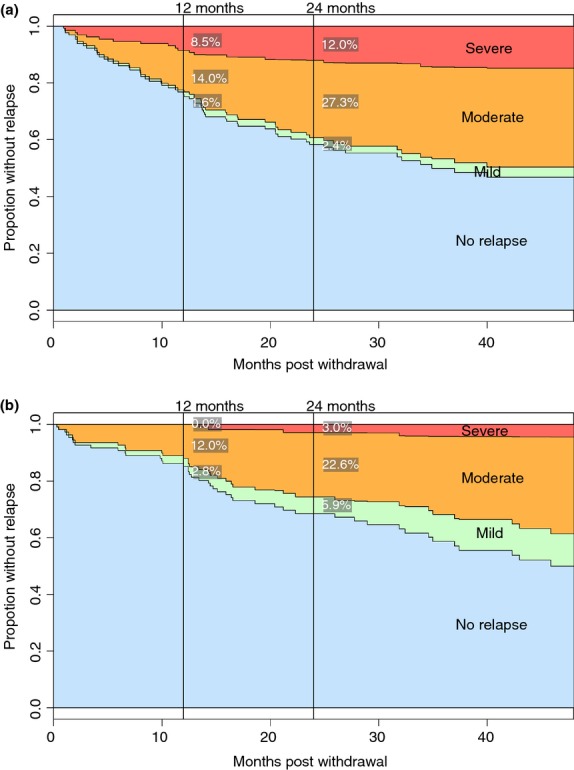
Survival analysis of relapse following withdrawal of thiopurines for sustained remission of Crohn's disease (a) and ulcerative colitis (b).

23% of CD patients had a moderate-to-severe relapse within 12 months of thiopurine withdrawal as compared to 12% in UC patients (Figure1). There was a significant difference in survival without moderate-to-severe relapse between CD and UC assessed by logrank test (*P* = 0.035). CRP at time of drug withdrawal was associated with significantly greater relapse in CD within 12 months (*P* = 0.005) but was not predictive in UC (Table 4). Relapse at 12 months in CD was also associated with having tapered the thiopurine at withdrawal (*P* = 0.004). In the UC cohort, white cell counts at withdrawal were significantly higher in those who relapsed by 12 months (*P* = 0.007), although the upper quartile was still in the normal range.

**Table 4:** Factors assessed against moderate-to-severe relapse by 12 months and diagnosis

	Crohn's disease			Ulcerative colitis		
	No relapse by 12 months	Relapse by 12 months	*P*	No relapse by 12 months	Relapse by 12 months	*P*
Female sex	62/96 (64.6%)	14/29 (48.3%)	0.174	34/90 (37.8%)	6/13 (46.2%)	0.783
Smoking status at withdrawal						
Current	17/92 (18.5%)	6/27 (22.2%)	0.366	4/82 (4.9%)	0/10 (0.0%)	0.825
Ex	15/92 (16.3%)	7/27 (25.9%)		24/82 (29.3%)	4/10 (40.0%)	
Never	60/92 (65.2%)	14/27 (51.9%)	54/82 (65.9%)	6/10 (60.0%)		
Age at diagnosis	24.0 (18.3–31.8)	25.5 (19.2–35.1)	0.587	28.0 (22.5–44.2)	28.0 (19.3–41.0)	0.586
Age when starting thiopurine	29.0 (21.3–41.0)	30.0 (22.5–43.0)	0.988	36.0 (26.5–52.5)	35.0 (27.0–44.0)	0.723
Additional reason for withdrawal	19/98 (19.4%)	2/29 (6.9%)	0.156	10/92 (10.9%)	2/13 (15.4%)	0.642
Maximum dose by weight (mg/kg)	1.8 (1.5–2.2)	1.9 (1.6–2.2)	0.39	1.9 (1.7–2.1)	2.1 (2.0–2.2)	0.101
Tapered at withdrawal	27/98 (27.6%)	17/29 (58.6%)	**0.004**	34/92 (37.0%)	7/13 (53.8%)	0.387
5ASA at withdrawal	26/98 (26.5%)	12/29 (41.4%)	0.193	71/92 (77.2%)	9/13 (69.2%)	0.504
Montreal location						
L1 ± L4	25/94 (26.6%)	4/27 (14.8%)	0.096			
L2 ± L4	30/94 (31.9%)	16/27 (59.3%)				
L3 ± L4	37/94 (39.4%)	7/27 (25.9%)				
Pure L4	2/94 (2.1%)	0/27 (0.0%)				
Montreal behaviour						
B1	66/93 (71.0%)	20/28 (71.4%)	1.000			
B2	12/93 (12.9%)	4/28 (14.3%)				
B3	15/93 (16.1%)	4/28 (14.3%)				
Montreal extent						
E1				19/85 (22.4%)	4/10 (40.0%)	0.276
E2				22/85 (25.9%)	3/10 (30.0%)	
E3				44/85 (51.8%)	3/10 (30.0%)	
Haemoglobin (g/L)*	151 (145–159)	143 (139–154)	0.101	149 (139–155)	145 (140–151)	0.496
White cell count (×10^9^/L)	6.2 (5.3–8.2)	7.6 (5.5–8.6)	0.270	5.9 (4.7–6.8)	7.7 (6.5–9.4)	**0.007**
Platelets (×10^9^/L)	265 (220–316)	268 (226–375)	0.303	260 (213–312)	290 (250.5–324)	0.218
CRP (mg/L)	4.0 (2.1–6.0)	7.0 (3.8–16.5)	**0.005**	2.5 (2.0–4.0)	3.0 (2.8–4.5)	0.286
Albumin (g/L)	44.0 (41.0–46.0)	43.0 (41.0–45.0)	0.259	45.0 (42.2–47.0)	44.0 (41.0–45.0)	0.187

* Haemoglobin for females scaled to male range to allow comparison across sexes.

*P* values less than 0.05 are highlighted in bold.

### Multivariable analysis

**Figure 2:**
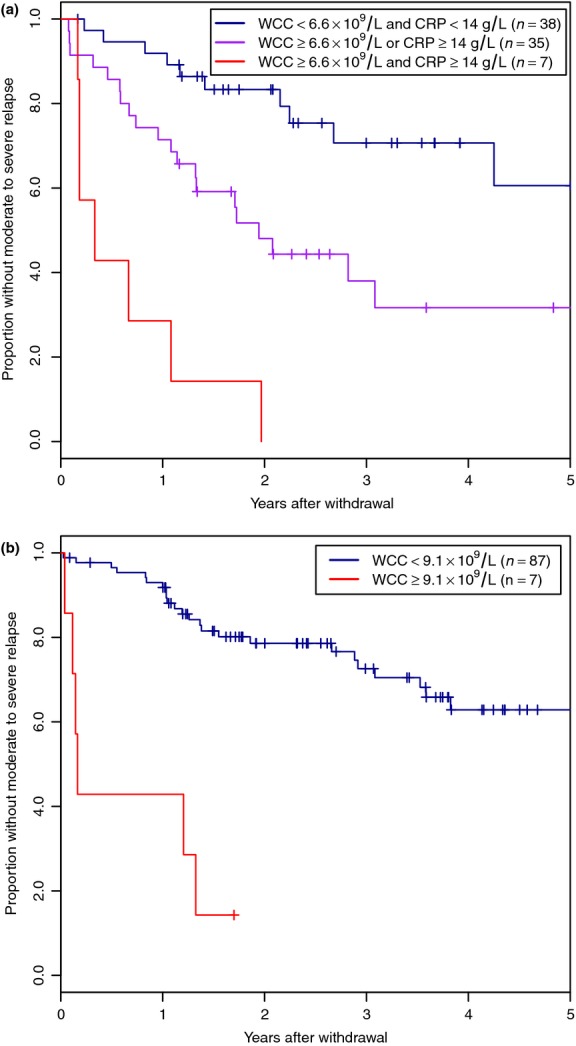
Survival analysis of relapse following withdrawal of thiopurines for sustained remission stratified by predictive factors in Crohn's disease (a) and ulcerative colitis (b).

Disease location and the most significant univariable laboratory parameters (haemoglobin, white cell count and CRP) were included in multivariable models for CD and UC. The Cox proportional hazards method was used to create a model to assess the contribution of each variable to risk of relapse. After backwards stepwise exclusion of variables that did not contribute to the model, WCC and CRP remained for CD, and only WCC remained for UC (Table5). Thresholds were then found to allow stratification of patients at higher and lower risk, and to allow creation of survival curves (Figure2).

**Table 5:** Multivariable analysis of predictive factors for relapse following thiopurine withdrawal: final Cox proportional hazards model. (a) Crohn's disease; (b) Ulcerative colitis

Analysis as continuous variables			Analysis as categorical variables		
Variable	Hazard ratio (95% confidence interval)	*P*-value	Optimum threshold to split data	Hazard ratio when split by threshold (95% CI)	*P*-value when split by threshold
(a)					
White cell count	1.18 (1.04–1.33)	0.011	≥6.6 × 10^9^/L	3.75 (1.87–7.54)	0.0002
C-reactive protein	1.04 (1.00–1.07)	0.035	≥14 g/L	3.2 (1.48–7.05)	0.003
(b)					
White cell count	1.44 (1.11–1.87)	0.007	≥9.1 × 10^9^/L	6.70 (1.86–24.2)	0.004

### Consequences of relapse

Among all CD patients, by 12 months, 23 patients (18%) had required systemic corticosteroids, four patients (3%) had required anti-TNF therapy, seven patients (5%) had required hospital admission and five patients (4%) had required resectional surgery. Among all UC patients, by 12 months, six patients (6%) had required systemic steroids, one patient (1%) hospitalisation and no patient required anti-TNF or resectional surgery. By the end of follow-up, a further three CD and two UC patients had required resectional surgery, although this was for dysplasia in one of the UC cases.

Within the 48 CD patients with a moderate-to-severe relapse at any point and did not require surgery or anti-TNF, 42 (88%) had a thiopurine re-introduced. Of those, reintroduction was successful in 31 (74%) although the majority (21/31, 68%) also required systemic steroids to reinduce remission. For UC patients, thiopurines were reintroduced in 24 of 34 (71%) patients with a moderate-to-severe relapse not requiring surgery or anti-TNF. This was successful in 22 patients (92%), with 11 (50%) requiring systemic steroids also.

## Discussion

In patients with CD, our study shows a moderate-to-severe relapse rate of 24% at 1 year and 39% at 2 years after thiopurine withdrawal. This is similar to published series showing a relapse rate of 21–41% with a cumulative increase in the rate with time (Table1). In addition, our study demonstrates a significant greater relapse rate in CD patients compared to UC. While this study does not address the rate of flare in those continuing therapy, a recent meta-analysis showed that the odds ratio of a flare in those stopping azathioprine versus those continuing was 0.15 [95% Confidence Interval (CI) 0.05–0.44] at 12 months and 0.30 (95% CI 0.08–1.23) at 18 months.^25^

Previous studies have shown that CRP of 20 mg/L or higher, a neutrophil count of 4.0 × 10^9^/L, a haemoglobin (<12 g/dL), male gender, age ≤31 and duration of remission less than four years were factors predictive of relapse.^17^,^18^ However, duration of AZA/MP use and the definition of remission varied with each study making it difficult to compare their study outcomes. Fraser *et al*., with *n* = 222 patients (79 CD, 143 UC), found no correlation between disease flare and clinical or laboratory indices.^9^ Our study shows that CRP is highly predictive of relapse in this cohort, a finding similar to Lemann *et al*.^19^ Tapering of thiopurine prior to withdrawal was also noted here to be associated with relapse, but practice with relation to tapering was quite different between the included centres and it is likely the observed differences in relapse rates relate to other, unmeasured factors rather than tapering itself.

In patients with UC, our study showed a lower relapse rate of 11% at 12 months and 21% at 24 months. This contrasts with a relapse rate after drug withdrawal in other published studies as high as 35–77% at 1 year and 65–75% at 5 years (Table1). We used strict criteria to define sustained remission which included continuous thiopurine use for a minimum ≥3 years and subsequent withdrawal when in sustained clinical remission (absence of symptoms and no corticosteroids for >6 months). This will have impacted the subsequent relapse rates.

In the UC cohort, our study shows that a raised white cell count is highly predictive of a relapse after drug withdrawal. Hawthorne *et al*. performed a small RCT trial and found younger age to be the statistically significant predictive factor for relapse.^24^ Cassinoti *et al*. performed a multicentre observational study of 127 UC patients and found that relapse during treatment with AZA, withdrawal of AZA due to drug toxicity and disease extent to be predictive of disease relapse at drug withdrawal. Patients in this study had concomitant aminosalicylates, masking the true effects of AZA.^23^ On the contrary, a large single centre study with 143 UC patients did not show any factors predictive of relapse.^9^

The definition of clinical remission is important when evaluating drug withdrawal studies. Studies have used various clinical disease activity indices and laboratory markers to define clinical remission. Two randomised controlled trials used the Crohn's Disease Activity Index (CDAI),^19^,^21^ while others used the Harvey Bradshaw Index (HBI).^9^,^17^,^20^ O'Donoghue *et al*. and Lobel *et al*. utilised the Physician Global Assessment (PGA) score to define remission and disease flare in CD.^3^,^22^ We used the PGA clinical index and corticosteroid use (in the last 6 months) to define remission.

Recapture data have only been reported by Treton *et al*. in CD patients where 22 of the 23 patients were successfully retreated with AZA.^18^ Although a small cohort, our study is the only study to show retreatment success in both disease groups. However, it should be noted that 25/29 patients with CD and moderate-to-severe relapse within 12 months (20% of the overall CD cohort) required systemic steroids, anti-TNF or hospital admission and five of these patients required resectional surgery. Further large studies are needed to ascertain re-treatment success as this would have an impact on our decisions to withdraw thiopurines.

With high cumulative relapse rates after thiopurine withdrawal in sustained remission, devising a set of key relapse indicators that encompass clinical, endoscopic and laboratory markers would be beneficial. Our study highlights the importance of risk stratification in patients before considering drug withdrawal. The knowledge of these predictive factors may be translated onto the anti-TNF group of patients; however, multicentre trials are required to validate this. The STORI study, looking at infliximab withdrawal in CD remission showed that the presence of no more than two risk factors (a combination of clinical and biological markers) carried a 15% risk of relapse at 1 year.^26^ Similarly, risk stratification in patients on long-term AZA/MP treatment who are risk of disease flare post drug withdrawal can be adopted in clinical practice. Future studies could use pre-defined risk groups to assess relapse rates post drug withdrawal.

In addition to risk factors for disease relapse, adverse events with long-term use must be taken into account when considering drug withdrawal. The small but definite association of non-melanoma skin cancer and lymphoma with long-term thiopurine use has been reported.^15^,^16^ The risk of non-melanoma cancer is greater when treating older patients with IBD.^15^ In addition, the comorbidity rate is significantly higher in the elderly group (age >65 years) with IBD.^27^,^28^ With an ageing population worldwide, the number of older patients with IBD is also expected to increase.^29^ Therefore, treatment strategies with thiopurines would need further evaluation and a careful consideration.

The key strengths of our study are threefold. Our study is one of the largest to date looking at AZA/MP withdrawal. While many studies used varied parameters to define remission, we used strict clinical parameters with at least 3 years of continuous thiopurine use prior to drug withdrawal.

Patients within this study were selected for withdrawal by their physicians on the basis of their assessment, and so the withdrawal rates may not be generalisable to all patients in clinical remission. For example, physicians may have been less likely to withdraw patients with perianal disease or rectal disease. There were also limited data available on faecal calprotectin; it is likely that as an accurate marker of endoscopic disease activity^30^ it would prove highly useful in predicting relapse in this context, as has been seen for infliximab withdrawal in the STORI study.^26^ The study may have been underpowered to fully assess the predictive power of all of the factors assessed.

Thiopurines remain an integral part of disease management in IBD patients with evidence of its role in sustaining long-term remission. However, bearing in mind the side effects and risks of malignancy with long-term immunosuppression, it is crucial to identify a sub-cohort who are at highest risk of disease flare. Our study and data from the STORI trial suggest that patients in clinical and biochemical remission have a low risk of relapse. Of those who relapse after drug withdrawal, reintroduction of thiopurines allows recapture in the majority of IBD patients, particularly in UC. However, in a select group of patients (CD cohort), long-term thiopurines may be in their best interest, especially if the consequences of disease flare have an impact on morbidity and subsequent remission rates.

## Authorship

*Guarantor of the article*: Dr Charlie Lees.

*Author contributions*: NAK and CWL conceived the study. PI, JM, MP, TA, JRFC, IA, JS, AJL, MS, JOL, CWL co-ordinated data collection at their respective sites. BW, CJG, RM, SR, RD, NH, RF, SM, SMS, CAL, HAH, DG collected the data. NAK aggregated the data and performed analysis. RK wrote the initial draft of the manuscript. NAK and CWL co-ordinated revision of the manuscript as guided by all of the authors who approved the final version of the manuscript.

## Supporting Information

**Table S1.** Reasons for exclusion from primary cohort. **Table S2.** Numbers of included patients from each centre. **Table S3.** Reasons for thiopurine withdrawal in addition to sustained remission in Crohn's disease and ulcerative colitis.
