# Supplementary material for: Thiopurine withdrawal during sustained clinical remission in inflammatory bowel disease: relapse and recapture rates, with predictive factors in 237 patients
Source: Aliment Pharmacol Ther. 2014 Oct 6;40(11-12):1313–23. doi: 10.1111/apt.12980 (PMC4232866; doi:10.1111/apt.12980)
Supplement: Supplementary file 1 [file apt0040-1313-sd1.docx]

## Supplementary table 1 – Reasons for exclusion from primary cohort

| Reason | Number (%) |
| --- | --- |
| Less than 12 months since withdrawal | 8 (3.0%) |
| Less than 35 months on thiopurine | 6 (2.3%) |
| IBD unclassified | 4 (1.5%) |
| Anti-TNF therapy at withdrawal | 4 (1.5%) |
| No information supplied on relapse | 3 (1.1%) |
| Drug withdrawn for inefficacy | 1 (0.4%) |
| Steroids at withdrawal (though for lymphoma) | 1 (0.4%) |
| Total | 27 (10.2%) |

Numbers shown as percentage of originally submitted cohort (263 patients)

## Supplementary table 2 – Numbers of included patients from each centre

| Centre | Patients with Crohn’s disease | Patients with ulcerative colitis | Total |
| --- | --- | --- | --- |
| Western General Hospital, Edinburgh | 43 | 27 | 70 |
| Royal Sussex County Hospital, Brighton | 13 | 18 | 31 |
| Royal Hallamshire Hospital, Sheffield | 16 | 14 | 30 |
| Barts and the London Hospital, London | 9 | 15 | 24 |
| Southampton General Hospital, Southampton | 15 | 8 | 23 |
| Royal Devon and Exeter Hospital, Exeter | 9 | 11 | 20 |
| Addenbrooke's Hospital, Cambridge | 4 | 9 | 13 |
| Royal Victoria Infirmary, Newcastle | 10 | 0 | 10 |
| Guy’s and St Thomas’ Hospitals, London | 5 | 2 | 7 |
| Glasgow Royal Infirmary, Glasgow | 5 | 3 | 8 |
| Sandwell and West Birmingham Hospitals, Birmingham | 0 | 1 | 1 |
| Total | 129 | 108 | 237 |

## Supplementary Table 3: Reasons for thiopurine withdrawal in addition to sustained remission in crohn’s disease and ulcerative colitis

| Reason | Crohn’s disease  (n=129) | Ulcerative Colitis  (n=108) |
| --- | --- | --- |
| **Sustained remission alone** | 107 (82.9%) | 95 (88.0%) |
| **Pregnancy** | 10 (7.8%) | 1 (0.9%) |
| **Infection** | 4 (3.1%) | 2 (1.9%) |
| **Leucopenia** | 1 (0.8%) | 3 (2.8%) |
| **Thrombocytopenia** | 1 (0.8%) | 1 (0.9%) |
| **Nodular regenerative hyperplasia** | 2 (1.6%) |  |
| **Unspecified side effects** | 2 (1.6%) |  |
| **Benign tumour** | 1 (0.8%) |  |
| **Lung cancer** | 1 (0.8%) |  |
| **Abnormal LFTs** |  | 3 (2.8%) |
| **Haematological malignancy** |  | 1 (0.9%) |
| **Warts** |  | 1 (0.9%) |
| **Vomiting in pregnancy** |  | 1 (0.9%) |
